# Autoinflammatory Keratinization Diseases (AiKDs): Expansion of Disorders to Be Included

**DOI:** 10.3389/fimmu.2020.00280

**Published:** 2020-02-21

**Authors:** Masashi Akiyama

**Affiliations:** Department of Dermatology, Nagoya University Graduate School of Medicine, Nagoya, Japan

**Keywords:** hidradenitis suppurativa, keratosis lichenoides chronica, pityriasis rubra pilaris, porokeratosis, pustular psoriasis

Inflammation caused by the hyper-activation of innate immunity due to genetic factors occasionally leads to inflammatory keratinization diseases of the skin. Such inflammatory keratinization diseases with genetic autoinflammatory pathomechanisms are called “autoinflammatory keratinization diseases” (AiKDs) (1). AiKDs also include disorders with combined pathological mechanisms of autoimmunity and autoinflammation. AiKDs possess primary genetic causal features associated with autoinflammation mainly in the epidermis and in the superficial dermis. Autoinflammation in these regions results in hyperkeratosis in the skin, leading to other cutaneous inflammatory symptoms of AiKDs (1, 2). The clinical phenotypes of AiKDs are variable, and each disease has unique characteristic manifestations, although common clinical features are hyperkeratotic lesions with inflammation (Table 1). Most AiKD patients have recurrent and persistent cutaneous lesions.

**Table 1:** Inflammatory keratinization diseases classified as AiKDs to date [adopted and modified from Akiyama et al. (1, 2)].

**Disease**	**Genetic causative or predisposing factor (frequency)**	**Pathogenic mechanism/pathway of inflammation in keratinocytes**
Pustular psoriasis and related disorders		
Generalized pustular psoriasis (GPP)	*IL36RN* mutations (predominant in early-onset cases and/or cases without psoriasis vulgaris)	IL36Ra↓ → IL-36↑ → MyD88↑ → NFκB/MAPK↑ → TNF, IL-1, IL-8, IL-17, IL-36, CXCL1, CXCL2, CCL20↑
	*CARD14* variants (occasional)	CARD14↑ → NFκB↑ → IL-36, IL-8, CXCL1, CXCL2, CCL20↑
impetigo herpetiformis	*IL36RN* mutations (predominant)	IL36Ra↓ → IL-36↑ → MyD88↑ → NFκB/MAPK↑ → TNF, IL-1, IL-8, IL-17, IL-36, CXCL1, CXCL2, CCL20↑
acrodermatitis continua	*IL36RN* mutations (occasional)	
palmoplantar pustular psoriasis (palmoplantar pustulosis)	*CARD14* variants (occasional)	CARD14↑ → NFκB↑ → IL-36, IL-8, CXCL1, CXCL2, CCL20↑
Pityriasis rubra pilaris (PRP)		
PRP type V	*CARD14* mutations (predominant)	CARD14↑ → NFκB↑ → IL-36, IL-8, CXCL1, CXCL2, CCL20↑
PRP other types	*CARD14* variants (infrequent)	
Keratosis lichenoides chronica (familial)	*NLRP1* mutation (unknown)	NLRP1↑ → inflammasome↑ → caspase-1↑ → IL-1↑ → TNF, GM-CSF, IL-36↑
Hidradenitis suppurativa (HS)	mutations in γ-secretase genes, *NCSTN, PSENEN* and *PSEN1* (infrequent)	Hypothesis: γ-secretase↓ → Notch signal↓ → aberrant differentiation and proliferation of the epidermis and the hair follicle epithelium
porokeratosis	mutations in mevalonate pathway-related genes, *MVK, MVD, PMVK* and *FDPS* (predominant)	Hypothesis: MVK, MVD, PMVK, FDPS↓ → geranylgeranyl diphosphate↓ → isoprenylated proteins (RAS, lamin B, etc.) ↓ → aberrant differentiation and proliferation of epidermal keratinocytes

Initially, AiKDs encompassed generalized pustular psoriasis (GPP), pityriasis rubra pilaris (PRP), and familial keratosis lichenoides chronica (KLC) (1, 2). It remains controversial as to whether GPP should be considered an AiKD. Many patients with GPP, acrodermatitis continua, or impetigo herpetiformis have been reported to have *IL36RN* mutations (3–9). GPP shows massive neutrophil infiltration leading to pustulosis and occasionally erosive skin. The level of hyperkeratosis is variable, depending on the timing, lesions and cases. However, keratinocyte differentiation and proliferation are thought to be frequently affected in the lesional epidermis of GPP, and GPP is considered to be a disease in which keratinization is affected, although hyperkeratosis is not apparent in some cases. In this context, it is reasonable to consider GPP a keratinization disease. In some GPP patients, rare mutations of both *IL36RN* and *CARD14* have been identified (8). This observation indicates that GPP is not caused by monogenic factors of either *CARD14* or *IL36RN*, but that these two genes are quite strong risk factors for GPP onset. Together with the strong HLA-class II associations with GPP, GPP seems to be a polygenic disease, with strong effects from *IL36RN* mutations. Mouse model results also support this view: *Il36rn*-null mice never show the disease spontaneously, but need imiquimod as a potential trigger to show GPP-like phenotypes (10). *IL36RN* mutations are occasionally found in GPP patients with psoriasis, although GPP patients without psoriasis seem to have *IL36RN* mutations more frequently (4, 9). In addition, Arakawa et al. (11) reported that induced *IL36RN* levels are lower in both GPP patients with and without *IL36RN* mutations, and proposed “*IL36RN* insufficiency” in GPP. Consistently, GPP patients can be successfully treated by IL36 signal blockade whether or not *IL36RN* mutations are present (12). Overall, it seems that the majority of GPP patients have hyperactivation in IL36 signaling. On the other hand, the evidence for autoimmunity in GPP is quite strong (13). Antigen-specific CD4^+^ T cell activation has been detected for certain HLA-class II alleles (11). In fact, treatments targeting T-cells are effective against GPP. However, in our initial definition of AiKDs, we defined the concept of AiKDs as encompassing diseases with mixed pathomechanisms of autoinflammation and autoimmunity (1). In cases of GPP, autoinflammation plays a significant role, although autoimmunity is also important. Thus, GPP is thought to be included in AiKDs.

Furthermore, both the *CARD14* pathway and the *IL36RN* pathway are associated not only with GPP, but also with psoriasis vulgaris. GPP and psoriasis vulgaris are thought to belong to the same disease entity on a spectrum. Indeed, *CARD14* mutations are associated with both psoriasis vulgaris and GPP, with the strongest CARD14 activity causing GPP (14, 15). Psoriasis genome-wide association studies show *CARD14* to be a risk gene for psoriasis (16), meaning that a CARD14-mediated pathway could be working in psoriasis patients without *CARD14* mutations. On the other hand, antigen-specific CD8^+^ T cell activation occurs in both psoriasis vulgaris and GPP (11). Together with identified autoantigens that activate CD8^+^ T cells in psoriasis (17, 18), this CD8^+^ T cell activation may represent a common psoriasis pathway that is also observed in GPP. Thus, psoriasis vulgaris also has both autoinflammatory and autoimmune pathogeneses, so it might well be included in AiKDs, because according to its initial definition, AiKDs encompass diseases with mixed pathomechanisms of autoinflammation and autoimmunity (1).

Most PRP type V patients harbor *CARD14* mutations (19) and are categorized as having an AiKD, and a small number of PRP cases of other types have *CARD14* variants and are also included in AiKDs. Craiglow et al. (20) proposed the term “CARD14-associated papulosquamous eruption” to indicate psoriasis patients and PRP type V patients carrying *CARD14* mutations, and they suggested that psoriasis/PRP type V patients with *CARD14* mutations have characteristic features of early onset, predominant facial skin symptoms, and good response to ustekinumab treatment. Such psoriasis/PRP type V patients with *CARD14* mutations are considered to be typical AiKD cases. In addition, some cases with pustular psoriasis may be related to mutations in *AP1S3* coding adaptor-related protein complex 1, sigma-3 subunit (AP1S3) (21). The AP1S3 molecule is involved in autophagosome formation, especially in keratinocytes. Loss of function of AP1S3 is considered to result in defective autophagy and abnormal accumulation of p62. p62 is known to mediate NFκB activation. Thus, p62 accumulation is thought to lead to NFκB hyper-activation, up-regulation of IL-1 signaling, and overexpression of IL-36 (21). In this context, pustular psoriasis associated with AP1S3—including cases with GPP, acrodermatitis continua, and palmoplantar pustulosis—is recognized as an AikD.

Recently, it has been proposed that hidradenitis suppurativa (HS), especially familial cases, be included in AiKDs. Mutations in the γ-secretase complex genes have been identified in patients and families with HS (22). HS patients carrying mutations in *NCSTN, PSENEN*, and *PSEN1* coding the γ-secretase complex show hyperkeratotic hair follicle epithelia in the infundibular region, resulting in the occlusion of hair follicles. Such occlusion is considered to be an essential part of the pathogenic mechanism behind HS (22). The plugging of the epidermis by keratinous materials is also a distinctive finding in skin lesions of PRP, a typical AiKD (19). Furthermore, it has been reported that tissue levels of TNF, caspase-1, IL-1, and IL-17 are high in the lesional skin of HS patients (23, 24). It might be reasonable to consider that HS, at least HS with γ-secretase gene mutations, is a clinical entity included in AiKDs because the primary step in the pathogenesis of HS is thought to be hyperkeratosis of the follicular epithelium in the infundibular region due to the aberrant activation of innate immunity. Indeed, occasionally, HS is seen in patients with autoinflammatory disorders including pyoderma gangrenosum and pyogenic sterile arthritis as autoinflammatory syndromes, such as pyoderma gangrenosum, acne and pyogenic arthritis (PAPA) syndrome, pyoderma gangrenosum, acne and HS (PASH) syndrome, and Majeed syndrome (25–29). HS and these autoinflammatory syndromes with HS might share the pathogenic mechanisms of hyperactivated innate immunity resulting in up-regulated production of IL-1 family cytokines and neutrophilic infiltration in the skin (26, 27). In considering the pathogenesis of HS, it is meaningful to recognize HS as an AiKD initiated by the hyperactivation of innate immunity in the epithelium of the follicular infundibulum. Heterozygous mutations in one of the three genes (*NCSTN, PSENEN*, and *PSEN1*) encoding the γ-secretase complex have been reported in HS (22). γ-secretase is a membrane-bound aspartyl protease complex which works in the intramembrane proteolysis of various membrane proteins, including Notch (28). Notch is a membrane receptor, and the binding of ligands to the extracellular portion of Notch triggers cleavage of the intracellular portion of Notch by γ-secretase, with the cleaved intracellular domain of Notch moving into the nucleus and regulating expression of various genes (29). In the skin, the differentiation and proliferation of epidermal cells, and the differentiation and maintenance of the epithelium of hair follicles and sebaceous glands are regulated by Notch signaling (22). Thus, reduced enzyme activity of γ-secretase complex due to mutations in *NCSTN, PSENEN*, and *PSEN1* is considered to down-regulate Notch signaling, resulting in aberrant differentiation and proliferation of the epidermis and the hair follicle epithelium in HS. The malfunction of γ-secretase was revealed to result in the abnormal differentiation of the epithelium in hair follicles via down-regulation of Notch signals in studies using model mice (22).

Porokeratosis encompasses diverse hyperkeratotic lesions consisting of single or multiple atrophic macules or plaques demarcated by hyperkeratotic ridges. Vertical columns of parakeratotic cells called “cornoid lamellae” are histological findings characteristic of porokeratosis (30). Porokeratosis is obviously a keratinization disease and also is regarded as an inflammatory disorder of the skin (30). In fact, significant inflammation is seen in eruptive pruritic papular porokeratosis, and cutaneous inflammatory disorders including localized cutaneous amyloidosis are complications of porokeratosis cases (30). We have proposed that porokeratosis be categorized as an AiKD. In 2012, mutations in *MVK*, one of the mevalonate pathway genes, were detected as a cause of disseminated superficial actinic porokeratosis (31). To date, four mevalonate pathway genes (*MVK, MVD, PMVK*, and *FDPS*) have been identified as causative of porokeratosis (30). The mevalonate pathway is known to produce isoprenoid precursors (30). Isoprenoids are precursors of various important molecules, such as heme A, cholesterol, and isoprenylated proteins (30). Isoprenylated proteins, including RAS and lamin B, regulate the growth and differentiation of cells (30). It was demonstrated that the deficiency of geranylgeranylpyrophosphate, a non-sterol isoprenoid in mevalonate pathway products, causes the activation of inflammasomes via the abnormal function of Rac1 (32). We speculate that malfunctions in the mevalonate pathway might result in the abnormal growth and differentiation of epidermal keratinocytes and autoinflammation in the lesions of porokeratosis. In this context, porokeratosis would be an AiKD, and it is occasionally hereditary (30). Indeed, one of the porokeratosis-causative mevalonate pathway genes, *MVK*, is also causative of hyperimmunoglobulinemia D and periodic fever syndrome, an established autoinflammatory syndrome (33). Biallelic mutations in *MVK* cause the more severe, systemic disease, hyperimmunoglobulinemia D and periodic fever syndrome, and heterozygous mutations in *MVK* lead to porokeratosis (31).

Porokeratosis patients with causative mevalonate pathway gene mutations have the mutations as heterozygous germline mutations. In skin lesions of porokeratosis, the expression of mutant alleles is higher than that of wild-type alleles, and the predominant expression of mutant alleles is considered to be a trigger for the formation of porokeratotic lesions (30). As a causative mechanism of the predominant expression of mutant alleles and the loss of function of wild-type alleles in porokeratotic lesions, an unelucidated DNA-methylation-independent epigenetic process is presumed in most cases (30), and in addition, genomic recombination which results in homozygosity of mutant alleles has been reported in other cases (30). Aberrant RNA editing was also reported in the lesional tissue of one porokeratosis case (30).

Concerning treatments for AiKDs, based on the autoinflammatory pathogenic mechanisms mentioned above, therapeutic strategies targeting molecules working in autoinflammatory cascades of pathomechanisms in AiKDs, such as cytokines, their receptors, and signal molecules, are expected to bring promising innovative therapies for various diseases included in AiKDs. Indeed, various treatments against pathogenic mechanisms are sufficiently effective for a number of AiKDs.

Successful treatments with granulocyte and monocyte adsorption apheresis (34), anakinra (an IL-1 receptor antagonist) (35), canakinumab (a monoclonal anti-IL-1β antibody) (36), infliximab (a monoclonal anti-human TNF-α antibody) (37), adalimumab (a monoclonal anti-TNF-α antibody) (38), ustekinumab (a monoclonal anti-human IL-12/IL-23 p40 antibody) (39), secukinumab (an anti-IL-17 monoclonal antibody) (40), and ixekizumab (a monoclonal anti-IL-17A antibody) (41) have been reported for GPP as an AiKD. Ustekinumab is also sufficiently effective against PRP with *CARD14* mutations (42). Concerning treatments for AiKDs due to *NLRP1* mutations, the IL-1 receptor antagonist anakinra was reported to be effective in cases of *NLRP1*-associated autoinflammation with arthritis and dyskeratosis (43). As for treatments for HS, adalimumab, infliximab, anakinra, and ustekinumab have been recommended (23).

The concept of AiKDs has ushered in a new era in inflammatory keratinization disorders. We assume that, in the near future, a growing number of inflammatory keratinization diseases may be recognized to be AiKDs, along with an advanced knowledge of novel pathomechanisms working in inflammatory keratinization disorders. The recognition of innate causative/predisposing issues and the precise assessment of their roles in disease etiology from the perspective of AiKDs promise to bring innovations that will afford more precise, targeted, causal therapies for various AiKDs.

## Author Contributions

The author confirms being the sole contributor of this work and has approved it for publication.

### Conflict of Interest

The author declares that the research was conducted in the absence of any commercial or financial relationships that could be construed as a potential conflict of interest.
